# Patterns and Trends of Polybrominated Diphenyl Ethers in Bald Eagle Nestlings in Minnesota and Wisconsin, USA

**DOI:** 10.1002/etc.5006

**Published:** 2021-03-10

**Authors:** William T. Route, Cheryl R. Dykstra, Sean M. Strom, Michael W. Meyer, Kelly A. Williams

**Affiliations:** ^1^ Great Lakes Inventory and Monitoring Network, US National Park Service Ashland Wisconsin USA; ^2^ Raptor Environmental, West Chester Ohio USA; ^3^ Wisconsin Department of Natural Resources Madison Wisconsin USA; ^4^ Wisconsin Department of Natural Resources Rhinelander Wisconsin USA; ^5^ Department of Biological Sciences Ohio University, Athens Ohio USA

**Keywords:** Bald eagle, Polybrominated diphenyl ether, Great Lakes, Mississippi River, Contaminants

## Abstract

We measured concentrations of up to 17 polybrominated diphenyl ethers (PBDEs) in plasma of 492 bald eagle (*Haliaeetus leucocephalus*) nestlings between 1995 and 2017 from 12 study areas in Wisconsin and Minnesota, USA. Geometric mean concentrations of the sum of 9 PBDE congeners (∑PBDE) measured across all years ranged from 2.88 to 10.8 µg/L, and nestlings in urban areas had higher concentrations than those in remote locations. Region‐wide from 2006 through 2017, we found that ∑PBDEs declined by 3.8% annually and congeners BDE‐47, ‐99, and ‐100 declined by 5.6 to 6.5%, whereas BDE‐153 and ‐154 had no significant declines. When categorized by waterbody type, nestlings from Great Lakes and river study areas had higher concentrations of ∑PBDEs than those at inland lakes, but river study areas spanned the extremes. From 2006 to 2017, ∑PBDEs declined by 7.3% annually in Great Lakes nestlings and by 3.2% in nestlings along rivers, and increased by 32.7% at inland lakes. Using a longer dataset (1995–2015), we found that ∑PBDEs declined in Lake Superior nestlings by 3.3% annually. Our results show that PBDEs declined in bald eagle nestling plasma in most study areas since PBDE production was reduced, but that concentrations remain high near urban centers and that trends differ by congener, study area, and waterbody type. *Environ Toxicol Chem* 2021;40:1606–1618. © 2021 The Authors. *Environmental Toxicology and Chemistry* published by Wiley Periodicals LLC on behalf of SETAC.

## Graphical abstract

As apex predators, bald eagles can serve as biosentinels of environmental contamination to aquatic environments. Blood plasma from bald eagle nestlings is particularly useful for measuring the extent of local contamination. PBDE = polybrominated diphenyl ether. For study area acronym abbreviations, see Table 1 footnote.

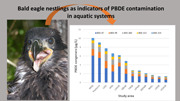

## INTRODUCTION

Polybrominated diphenyl ethers (PBDEs) were introduced in the 1970s as flame retardants, and their extensive use, persistence in the environment, and bioaccumulative properties have resulted in widespread contamination of water, wildlife, and humans (Hites 2004, 2006; Chen et al. 2010; Venier et al. 2015; Akortia et al. 2016). The toxic effects of PBDEs include disruption of thyroid and immune functions (Fernie et al. 2005, 2017) and possible deleterious effects on behavioral development and reproduction (Fernie et al. 2005; Henny et al. 2009; Klinsic et al. 2020). The PBDEs are chemically similar to polychlorinated biphenyls (PCBs), which were banned in the United States in the 1970s due to their toxicity (US Environmental Protection Agency 1979). Like PCBs, PBDEs could theoretically have up to 209 different congeners, which differ by the number and position of bromine atoms (Rahman et al. 2001). Unlike PCBs, the toxicity of individual PBDE congeners is not well understood, although there are greater concerns about the lower brominated congeners, in particular BDE‐47, in terms of persistence and toxicity (Costa et al. 2008; Souza et al. 2016).

The PBDEs have been used in 3 different congener formulations: penta‐BDE, octa‐BDE, and deca‐BDE. Penta‐ and octa‐BDE production was reduced globally starting in the early 2000s, largely ended in North America by 2004, and was banned internationally in 2009 under the Stockholm Convention (Li et al. 2015). Production of the higher brominated deca‐BDE formulations increased in the early and mid‐2000s to make up for penta‐ and octa‐BDE reductions, but manufacturing of deca‐BDEs was also phased out by 2013 (Venier et al. 2015). Li et al. (2015) estimated that approximately 1.3 to 1.5 million metric tonnes of PBDEs have been produced globally and that 90 to 95% of penta‐BDE was used in polyurethane foams for the automotive and upholstery industries; approximately 95% of octa‐BDE was used for acrylonitrile–butadiene–styrene polymers in the electronics industry; and 99% of deca‐BDE was used to retard flammability in a wide variety of plastics in commercial products such as computers, televisions, and other appliances. Concentrations of PBDEs in wildlife in some areas of North America leveled off or began decreasing in the early 2000s (Crimmins et al. 2012; Route et al. 2014a; Venier et al. 2015). Nonetheless, PBDEs are present in many products still in use, including textiles, plastics, insulation, and electronics, and their disposal and degradation continue to release PBDEs into the environment (de Wit 2002; Akortia et al. 2016). Atmospheric transport and deposition allow PBDEs to reach remote locations (Venier et al. 2015), and due to past contamination, lake and stream sediments (Li et al. 2006) and water from tributaries (Melymuk et al. 2014) also act as sources of PBDEs.

The PBDE burden in piscivorous fish and birds of the Midwestern United States is dominated by 5 congeners associated with penta‐BDE formulations, BDE‐47, ‐99, ‐100, ‐153, and ‐154 (Blocksom et al. 2010; Gandhi et al. 2017; Zhou et al. 2019), although other congeners are found. The exact ratio of PBDE congeners depends in part on the source, but can also vary by species (Gandhi et al. 2017; Su et al. 2017). Congener patterns of piscivorous birds like bald eagles (*Haliaeetus leucocephalus*) are typically dominated by BDE‐47, ‐99, and ‐100 (Henny et al. 2009; Chen et al. 2010). Bioaccumulation of PBDE congeners in aquatic wildlife can also vary regionally and among specific waterbodies, likely due to sources of contamination, atmospheric conditions driving air deposition, regional and local hydrology (e.g., drainage area), retention time of the aquatic system (flowing vs impoundment), and complexity of the food web (Dykstra et al. 1998, 2005a; Warnke et al. 2002; Gill and Elliott 2003; Zhu and Hites 2004; Elliot et al. 2009; Route et al. 2014a; Gandhi et al. 2017).

Persistent contaminants like PBDEs bioaccumulate as they transfer up trophic levels so that measuring them in tissues of apex predators is an effective method for understanding their distribution in the environment (Elliott and Norstrom 1998; Elliott and Harris 2001–2002; Hinck et al. 2009). For decades bald eagles have served as bioindicators of environmental contamination (Elliott and Norstrom 1998; Dykstra et al. 2001; Elliott and Harris 2001–2002; Wierda et al. 2016; Route et al. 2019), and nestlings are particularly useful because they reflect contamination within a 4‐ to 5‐km^2^ nesting territory (Garrett et al. 1993; Watson 2002). Monitoring of PBDE congener profiles over time in different waterbodies within a region can provide insight into sources and exposure pathways (Route et al. 2019) and levels of risk (Elliott et al. 2018).

To provide state and federal regulators with information on current PBDE levels, and to evaluate the effect of reducing PBDE production, we assessed patterns and trends in PBDE concentrations in bald eagle nestlings across a variety of waterbodies in Minnesota and Wisconsin (USA). We hypothesized that we would find a significant decline in PBDE concentrations in nestling plasma following the reduction in penta‐PBDE production by 2004. We further predicted that concentrations and trends would vary among study areas and waterbody type due to differences in sources and pathways into the prey fed to bald eagle nestlings.

## MATERIALS AND METHODS

### Study areas

**Figure 1:**
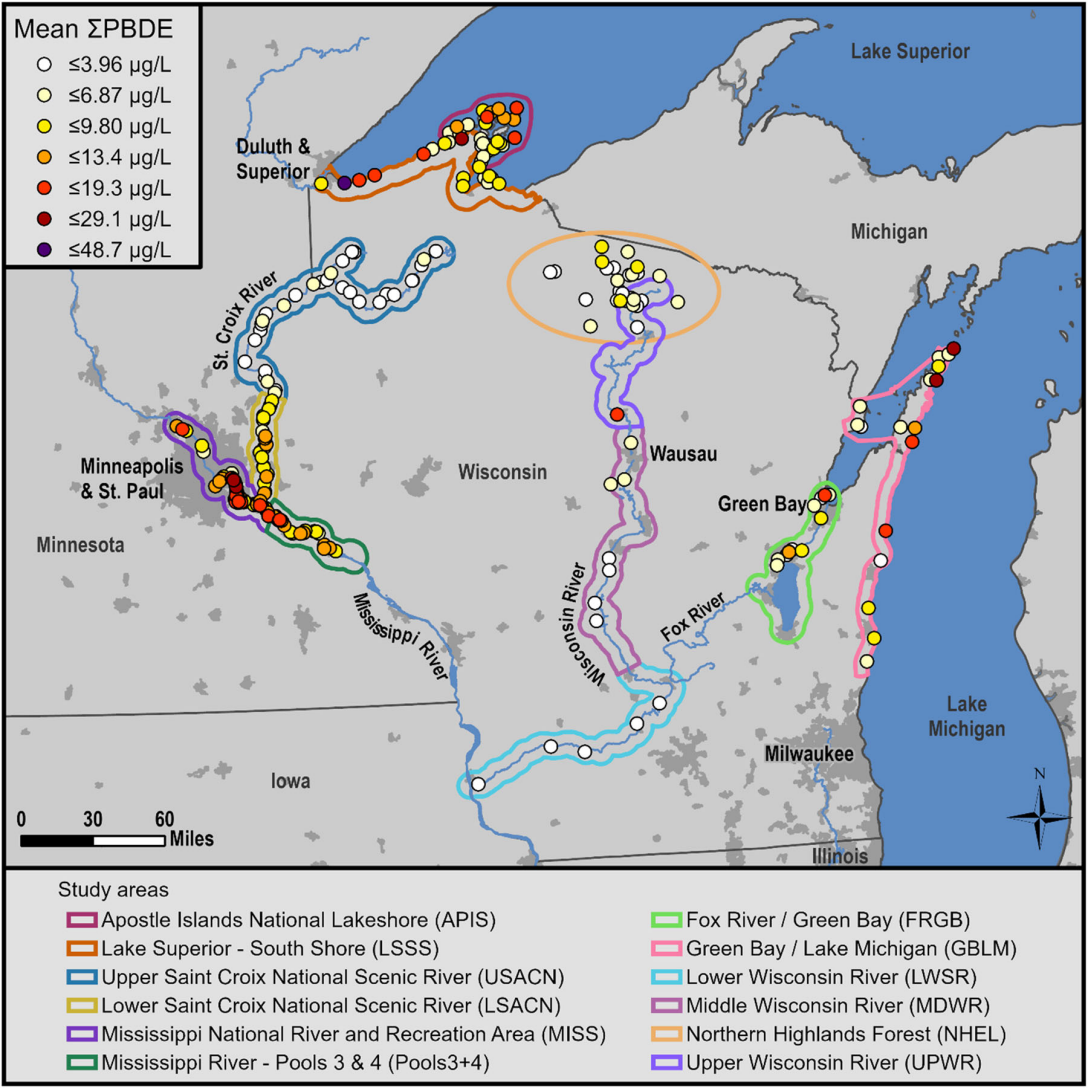
Geometric mean sum polybrominated diphenyl ether (∑PBDE) concentrations (µg/L) in bald eagle (*Haliaeetus leucocephalus*) nestling plasma at 241 nesting territories in 12 study areas in Minnesota and Wisconsin, USA. The scale for colored dots was determined using ArcMap natural breaks feature. Population centers of ≥5000 people are highlighted in dark gray. See the Supplemental Data, Figure SI1, for additional location, geographic, and population information.

We measured PBDE concentrations in blood plasma from nestling bald eagles at 12 study areas in Wisconsin and Minnesota (Supplemental Data, Figure SI1; see also Figure 1). Study areas included Apostle Islands National Lakeshore, Lake Superior's southern shore in Wisconsin, upper St. Croix National Scenic Riverway, lower St. Croix National Scenic Riverway, Mississippi National River and Recreation Area, Pools 3 and 4 of the Mississippi River (Pools3+4), Fox River and lower Green Bay, upper Green Bay and Lake Michigan, upper Wisconsin River, middle Wisconsin River, lower Wisconsin River, and Northern Highlands American Legion State Forest. We further classified plasma samples as coming from eagles residing on 1 of 3 waterbody types: Great Lakes (Apostle Islands, Lake Superior southern shore, Green Bay/Lake Michigan, and some nests from Fox River/Green Bay); rivers (Mississippi River, Pools3+4, upper St. Croix River, lower St. Croix River, upper Wisconsin River, mid‐Wisconsin River, lower Wisconsin River, and the remainder of nests from Fox River/Green Bay); or inland lakes (Northern Highlands). For Fox River/Green Bay, nests <8 km from shore were classified as Great Lakes and the remainder as river nests.

### Sample collection

Tree climbers accessed nests when nestlings were expected to be 5 to 9 wk old and lowered them to the ground. We measured and weighed nestlings, estimated age from length of the 8th primary (Bortolotti 1984), and determined sex either by genetic analysis based on polymerase chain reaction (Boutette et al. 2002) or by footpad length (Bortolotti 1984). We collected approximately 10 mL of blood from the brachial vein of at least one nestling from each nest and stored samples in a heparinized vacutainer on blue ice. At the end of each field day, we centrifuged samples and froze the plasma at −20 °C until analysis.

Two hundred eighty‐four samples in 6 study areas were previously analyzed and reported for PBDEs in 1995 to 2011 (Route et al. 2014a). In the present study we include 190 additional samples that extend through 2017, add 8 previously unreported PBDE congeners, and add 6 more study areas to elucidate larger regional patterns over a longer time span. Some of the additional samples had been stored frozen since as early as 1995 (1995–2001, *n* = 18; Supplemental Data, Table S1). However, the most abundant PBDE congeners (BDE‐47, ‐99, 100, and ‐153) have been found to be reasonably stable in frozen archives of fish tissue with loss rates of 1 to 2% annually over 10 yr (Batterman et al. 2007). Similarly, Custer et al. (2009) concluded that PBDE loss rates in frozen samples of great blue heron (*Ardea herodias*) egg albumen over 14 yr were likely insignificant based on measurements of chemically similar PCBs in the same frozen samples.

### Laboratory measurements

All samples were measured for 9 congeners (BDE‐28, ‐47, ‐66, ‐85, ‐99, ‐100, ‐138, ‐153, and ‐154). An additional 8 congeners (BDE‐49, ‐156, ‐183, ‐196, ‐197, ‐206, ‐207, and ‐209) were measured for samples collected from 2010 to 2017 and for archived samples from 1995 to 2001 (Supplemental Data, Table S1). All samples were measured by the Wisconsin State Laboratory of Hygiene (Madison, WI, USA; 2012) using a consistent method. The percent recovery of known quantities in quality assurance samples ranged from 80.2 to 100.9%, and the limits of quantification (LOQ) ranged from 0.2 to 10.0 µg/L (Supplemental Data, Table S2). Field and laboratory methods have been described in greater detail previously (Route et al. 2014a, 2019).

### Statistical analyses

For comparisons and trends by study area and waterbody type from 2006 to 2017, we included results for 9 congeners that were measured across all years (*n* = 474). Similarly, we used results for these 9 congeners to assess trends at 2 Lake Superior study areas from 1995 to 2015 (*n* = 166). The LOQ for the 9 congeners varied, and the percentage of congeners >LOQ ranged from 0.8 to 99.6% (Supplemental Data, Table S2). To account for results below the LOQ, we used the Kaplan–Meier technique to estimate means and totals (Helsel 2012). The results were not normally distributed, so we used either log‐normal or log_10_ transformations as appropriate for each analysis. All analyses were conducted in R Ver 3.5.1 (R Core Team 2017). Kaplan–Meier means were calculated in R with the function *cenfit* in the package NADA (Lee 2017) and then multiplied by the number of congeners for that sample.

### Sum of PBDEs

For study area comparisons of nestling plasma concentrations, we included summed geometric means of 9 congeners (hereafter termed ∑PBDE) measured in all study areas and years using mixed effects models (package nlme; Pinheiro et al. 2017). We log_10_‐transformed ∑PBDE to meet assumptions of normality. We used territory as a random effect because nestlings from the same territory from year to year usually have the same parents and feeding area (Garrett et al. 1993). We fit a set of models using maximum likelihood with either a random intercept or random slope and intercept, with study area, year, and their interaction as fixed effects. We used Akaike information criterion with correction for sample sizes (AIC*_c_*) to select the most supported model (Burnham and Anderson 2002; Zuur et al. 2009) with the function MuMIN (Barton 2019). We then refit that model with restricted maximum likelihood (REML) to avoid biased estimates (Zuur et al. 2009). We used Tukey's post hoc comparisons with the function emmeans (Lenth 2019) to compare study area differences. We back‐transformed parameter estimates (10^*β*^) to obtain geometric means and trends.

For samples grouped by waterbody type, we used the same mixed effect model technique, but also nested territory within study area to improve fit. We fit a set of models using maximum likelihood with a random intercept, with waterbody type, year, and their interaction as fixed effects, and then we reran the most supported model with REML. We again used Tukey's post hoc tests to sort differences and estimate trends.

### Individual congeners

In addition to ∑PBDEs, we compared 5 congeners (BDE‐47, ‐99, ‐100, ‐153, and ‐154) that generally have the highest concentrations in aquatic biota (Route et al. 2014a; Zhou et al. 2019). To estimate study area means, we fit a parametric survival mixed effect model (frailty model; Govindarajulu et al. 2011) using the function *survreg* in package survival for each analyte (Spears and Isanhart 2014). We assigned territory as a random effect with gamma distribution and an expectation maximization algorithm (method = “em”). A left‐censored survival object for each congener and the respective LOQ was created for each model. Models were fit with a log‐normal distribution, and residual plots were checked to ensure assumptions were met. We omitted nestling age because we found no effect of age. We did not use year with study area because of too few samples in several study areas; however, by pooling study areas, we had sufficient sampling to estimate trends by waterbody type. We used post hoc comparisons as described previously.

For congeners BDE‐47, ‐99, ‐100, ‐153, and ‐154, we tested the effect of waterbody type in separate analyses using the frailty model as just described. We used year in the models as a covariate; however, inland lakes had limited sampling among years. We used the emmeans function as just described for post hoc comparisons, and we report values on the response scale. Trends were back‐transformed using *e* ^*β*^ because a log‐normal distribution was used.

For 8 novel congeners, we report the geometric means and range for 3 that had >10% but <40% of samples above the LOQ, and we report only the percentage of samples above the LOQ for the remaining 5.

### Long‐term trends

We used a mixed effects model in the package nlme (Pinheiro et al. 2017) to examine long‐term trends of ∑PBDEs at Lake Superior southern shore and Apostle Islands (1995–2015). We log_10_‐transformed ∑PBDEs to meet assumptions of normality. As in the previous section, *Sum of PBDEs*, we used territory as a random effect, and study area, year, and nestling age as fixed effects.

## RESULTS

We analyzed 492 bald eagle nestling plasma samples from 241 nesting territories in 12 study areas across Wisconsin and Minnesota from 1995 to 2017 (Figure 1 and Supplemental Data, Table S1). Nestlings averaged 43.6 ± 0.4 d old (19–71 d), and weighed 3.57 ± 0.03 kg (1.80–5.50 kg), and the sex ratio was near parity (249 females: 243 males; χ^2^ = 0.073, *df* = 1, *p* = 0.79).

### PBDE concentrations

The ∑PBDEs of 9 congeners measured in all nestlings between 2006 and 2017 (*n* = 474) varied significantly among study areas (Figure 1 and Table 1) and were greatest at Mississippi River, an urban study area, and lowest at upper St. Croix River, a sparsely populated study area. When waterbody type was considered, nestlings on the Great Lakes and rivers had significantly higher ∑PBDEs than those on inland lakes (*t* = 3.52, *p* = 0.01; *t* = –3.24, *p* = 0.02, respectively), but nestlings on the Great Lakes and rivers were not significantly different from each other (*t* = 1.07, *p* = 0.5).

**Table 1:** Geometric means and (range) of sum polybrominated diphenyl ethers (∑PBDEs) and 5 congeners in nestling bald eagle (*Haliaeetus leucocephalus*) plasma from 2006 to 2017 at 12 study areas and 3 waterbody types in Wisconsin and Minnesota (sorted by ∑PBDE)

		Geometric means and (range) in µg/L^a^					
Study area (waterbody type)	No.	∑PBDE	BDE‐47	BDE‐99	BDE‐100	BDE‐153	BDE‐154
MISS (river)	139	10.8E (3.23–48.7)	6.10H (1.20–29.0)	1.47E (0.230–6.60)	1.37E (0.250–6.80)	0.590C (<LOQ–2.70)	0.570DE (<LOQ–2.40)
Pools3+4 (river)	33	9.72DE (6.31–20.6)	5.56H (3.30–11.0)	1.31E (0.480–3.40)	1.21CDE (0.830–2.40)	0.500BC (<LOQ–1.40)	0.500CD (<LOQ–1.10)
GBLM (Great Lake)	30	7.62CDE (2.25–31.1)	1.99DE (0.620–5.30)	1.07D (<LOQ–6.50)	0.810C (<LOQ–2.70)	0.550BC (<LOQ–4.20)	0.470BC (<LOQ–3.64)
LSSS (Great Lake)	16	8.36CDE (4.28–45.5)	3.25FG (1.10–10.0)	1.63E (0.420–15.0)	1.58E (0.420–6.80)	0.610BC (<LOQ–9.50)	0.700EF (<LOQ–2.20)
APIS (Great Lake)	58	7.86CD (3.38–37.2)	2.51EF (0.860–8.60)	1.52E (0.440–9.20)	1.21DE (0.330–6.60)	0.590C (<LOQ–5.60)	0.780F (<LOQ–2.80
LSACN (river)	65	7.79CD (3.18–23.7)	3.66G (0.820–14.0)	0.830D (LOQ–2.60)	0.900CD (LOQ–3.60)	0.400B (<LOQ–0.960)	0.410C (<LOQ–0.970)
FRGB^b^ (river/Great Lake)	16/7	5.88BC (2.70–16.2)	1.46CD (0.580–5.20)	0.490C (<LOQ–1.40)	0.480B (<LOQ–1.70)	0.210A (<LOQ–1.40)	0.140A (<LOQ–2.10)
UPWR (river)	8	4.79ABCD (1.51–15.2)	1.40C (0.320–7.60)	0.430BC (<LOQ–1.20)	0.380AB (<LOQ–1.40)	0.240A (<LOQ–0.780)	0.260AB (<LOQ–1.10)
MDWR (river)	7	4.61ABC (2.78–6.71)	1.30BC (0.490–3.80)	0.260AB (<LOQ–0.490)	0.190A (<LOQ–0.730)	0.110A (<LOQ–0.410)	0.230A (<LOQ–0.480)
NHEL (inland lake)	25	4.02AB (0–9.15)	0.830AB (<LOQ–3.40)	0.350AB (>LOQ–1.40)	0.270A (<LOQ–1.10)	0.100A (<LOQ–0.890)	0.150A (<LOQ–0.840)
LWSR (river)	5	2.98AB (2.27–3.08)	0.830ABC (0.670–0.960)	0.250ABC (LOQ–0.360)	0.180A (<LOQ–0.230)	<LOQ (<LOQ–0.150)	0.150A (<LOQ–0.480)
USACN (river)	65	2.88A (1.36–8.10)	0.630A (0.190–2.20)	0.240A (<LOQ–0.630)	0.230A (<LOQ–0.620)	0.150A (<LOQ–0.620)	0.180A (<LOQ–0.580)
Waterbody type:							
Great Lakes	111	7.18B (2.25–45.5)	2.35B (0.620–10.0)	1.08C (LOQ–15.0)	0.940C (LOQ–6.80)	0.450C (LOQ–9.50)	0.540C (LOQ–3.64)
Rivers^c^	338	5.85B (1.36–48.7)	2.38B (0.190–29.0)	0.750B (0.120–7.0)	0.610B (0.100–6.80)	0.320B (LOQ–2.70)	0.330B (LOQ–2.40)
Inland lakes	25	0.920A (0–9.15)	0.030A (0.150–3.40)	0.240A (LOQ–1.40)	0.200A (LOQ–1.10)	0.090A (LOQ–0.890)	0.140A (LOQ–0.840)

^a^ Geometric means with the same capital letter within columns are not significantly different (Tukey‐adjusted comparisons). All comparisons for ∑PBDE were significant at *p* < 0.01; for congeners BDE‐47, ‐99, and ‐100, they were significant at *p* < 0.001.

^b^ For the FRGB study area, 16 samples were categorized as river samples for analysis by waterbody type.

∑PBDE = sum polybrominated diphenyl ether; APIS = Apostle Islands; LSSS = Lake Superior southern shore; USACN = Upper St. Croix River; LSACN = Lower St. Croix River; MISS = Mississippi River; Pools 3 + 4 = Pools 3 and 4 of the Mississippi River; FRGB = Fox River/Green Bay; GBLM = Green Bay/Lake Michigan; UPWR = Upper Wisconsin River; MDWR = Mid‐Wisconsin River; LWSR = Lower Wisconsin River; NHEL = Northern Highlands; LOQ = limits of quantification.

Five congeners (BDE‐47, ‐99, ‐100, ‐153, and ‐154) had results >LOQ in >60% of samples (Supplemental Data, Table S2) and comprised the bulk of the PBDE burden, although concentrations differed between study areas (Table 1). Concentrations of BDE‐47 were highest at Mississippi River and Pools3+4 and lowest at upper St. Croix River, lower Wisconsin River, and Northern Highlands. We also found significant differences in BDE‐47 among waterbody types, with higher concentrations in Great Lakes and rivers compared with inland lakes. Concentrations of BDE‐99 were highest at Lake Superior southern shore and Apostle Islands and lowest at upper St. Croix River and lower Wisconsin River. Similarly, we found differences in BDE‐99 among waterbody types, with Great Lakes highest followed by rivers and inland lakes (all contrasts *p* < 0.001). The highest concentrations of BDE‐100 were at Lake Superior southern shore and Mississippi River and lowest at lower Wisconsin River and mid‐Wisconsin River study areas, and was highest in samples from the Great Lakes and rivers and lowest in inland lakes (second‐best model included only waterbody type; all contrasts *p* < 0.05). The study area lower Wisconsin River was dropped for the BDE‐153 analysis because all samples were <LOQ. Of the remaining areas, both BDE‐153 and ‐154 were highest at Lake Superior southern shore and Apostle Islands, whereas BDE‐153 was lowest at Northern Highlands and mid‐Wisconsin River, with BDE‐154 lowest at Fox River/Green Bay, and Northern Highlands. The greatest concentrations of both BDE‐153 and ‐154 were found in the Great Lakes, and these were significantly higher than in rivers and inland lakes (*p* < 0.001).

We did not assess in detail the concentrations of 12 PBDE congeners that had approximately >60% of samples <LOQ (range 59.7–100% < LOQ; Supplemental Data, Table S3). In particular, higher brominated congeners (BDE‐156–BDE‐209) had <2% of samples >LOQ, and the occurrence of these congeners were limited to the Green Bay/Lake Michigan study area. However, 3 of the 12 congeners (BDE‐28, ‐49, and ‐66) had >10% >LOQ, were found in 4 to 9 study areas, and reached levels as high as 0.750 µg/L (Pools3+4), 0.590 µg/L (lower St. Croix River), and 0.160 µg/L (Apostle Islands), respectively (Supplemental Data, Table S4).

### PBDE trends

**Figure 2:**
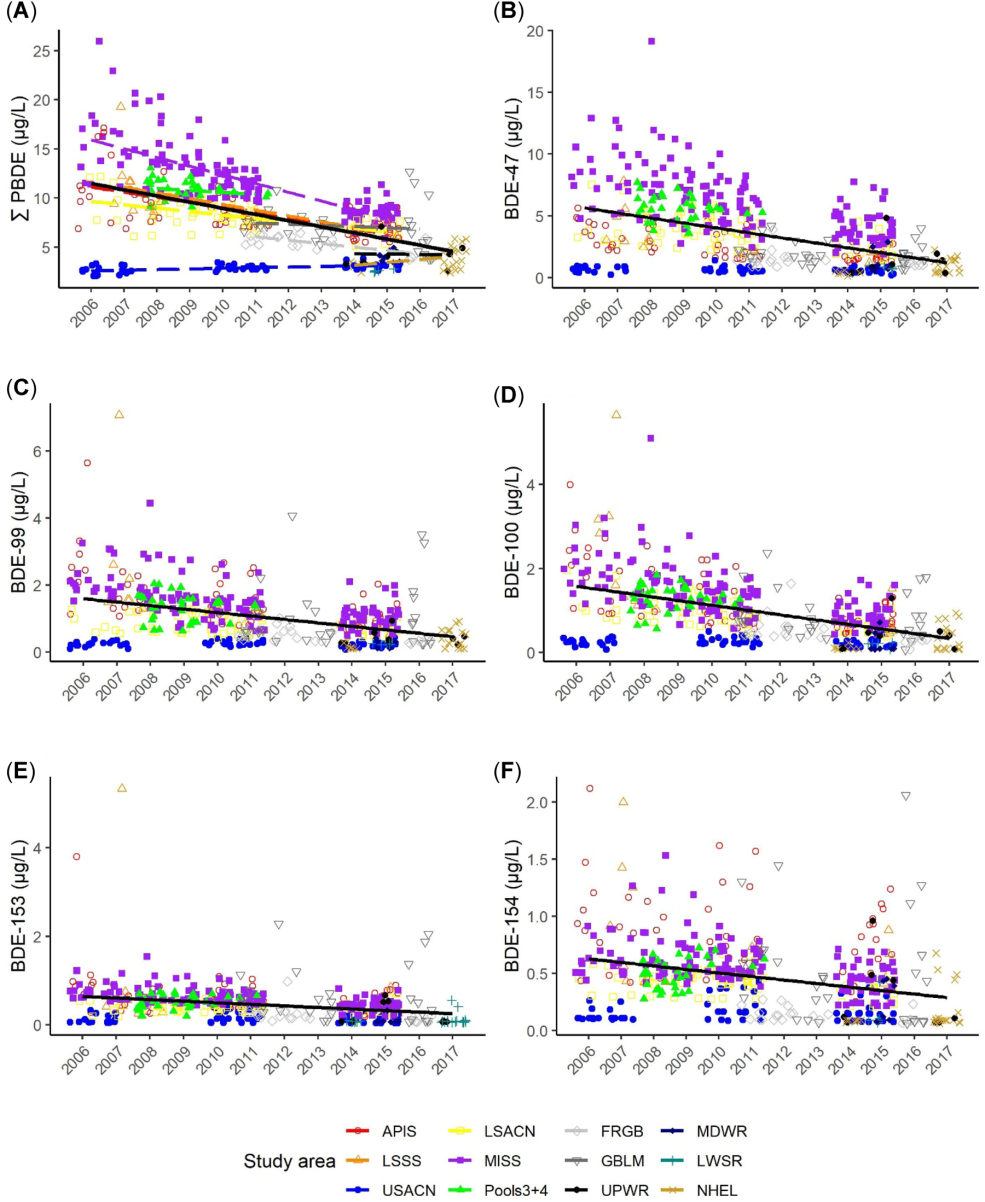
Trends in sum polybrominated diphenyl ethers (∑PBDEs) and 5 congeners in bald eagle (*Haliaeetus leucocephalus*) nestling plasma by study area and year. Bold black trend lines are for all study areas combined. Trends in (**A**), (**B**), (**C**), and (**D**) are significant (*p* < 0.01), and trends in (**E**) and (**F**) are not significant. For study area acronym definitions, see Table 1 footnote.

**Figure 3:**
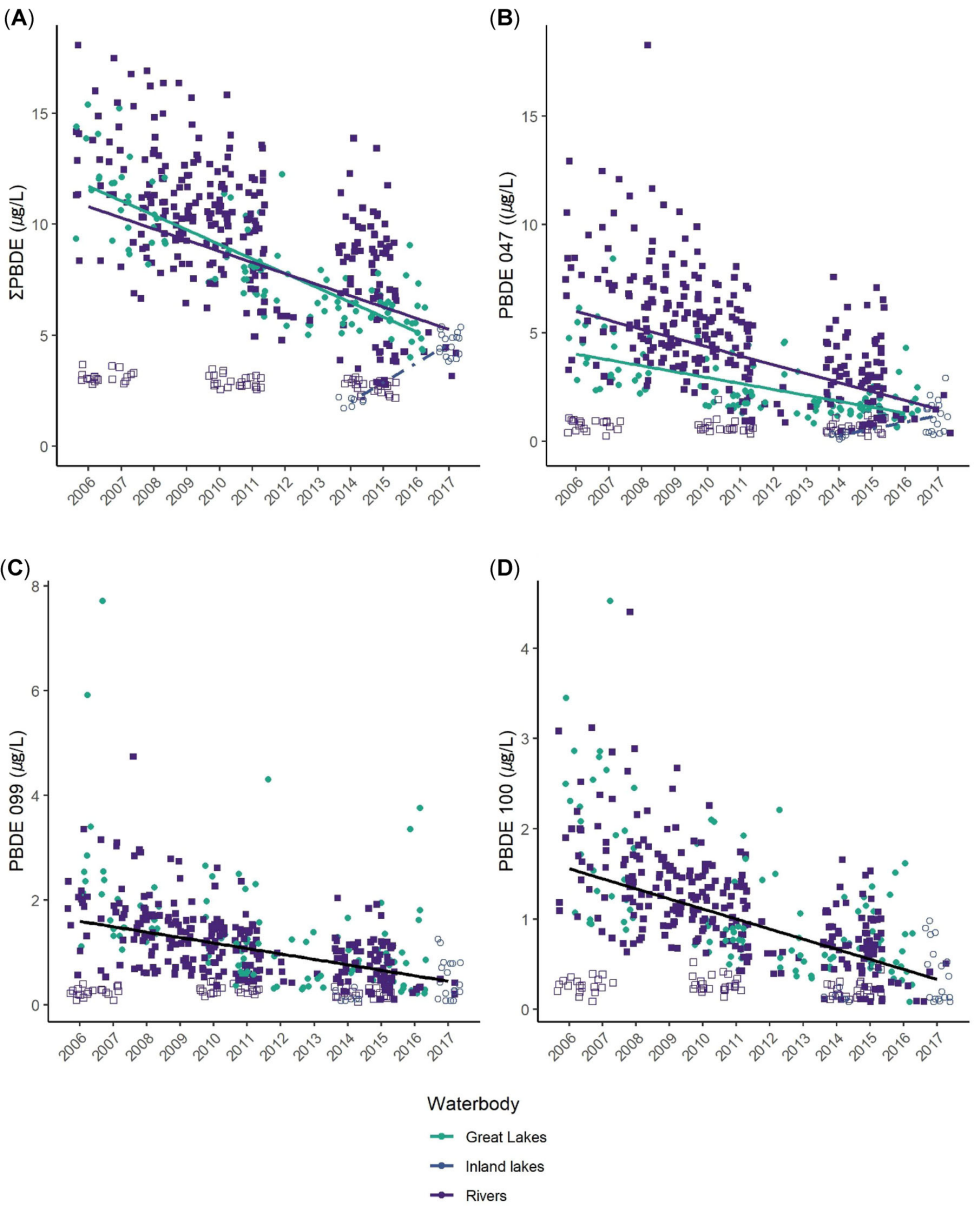
Trends in polybrominated diphenyl ethers (PBDEs) in bald eagle (*Haliaeetus leucocephalus*) nestling plasma by waterbody type and year. Filled green circles = Great Lakes; open circles = inland lakes; open purple squares = upper St. Croix River (USACN); filled blue squares = all other rivers. In (**A**) and (**B**), green trend line = Great Lakes, purple trend line = rivers, and dotted trend line = inland lakes; In (**C**) and (**D**) black trend line = all water types combined. We could not estimate trends for BDE‐153 or ‐154 due to poor model fit. All trend lines are significant at *p* < 0.01.

Considering all 12 study areas, we found a significant region‐wide decline of 3.8% annually in ∑PBDEs (model included territory as a random effect; study area and year as fixed effects, AIC wt = 0.99; slope (*β* = –0.0168, *p* = 0.0001; Table 2 and Figure 2). When we grouped by waterbody type, we observed a 7.3% annual decline for Great Lakes nestlings (*β* = –0.0329; *t* = –5.19, *p* < 0.001); a 3.2% decline for nestlings from rivers (*β* = 0.0185, *t* = 2.49, *p* = 0.01); and an increase of 32.6% for inland lakes (*β* = 0.1557, *t* = 5.13, *p* < 0.001), although the inland lakes finding should be considered with caution because that study area had few samples (*n* = 25) and was sampled only in 2014 and 2017 (Figure 3).

**Table 2:** Summary of trends in PBDEs in bald eagle (*Haliaeetus leucocephalus*) nestling plasma for all study areas combined, categories of waterbody type, and long‐term trends at Lake Superior (%)

	Water type	∑PBDEs	BDE‐47	BDE‐99	BDE‐100	BDE‐153^a^	BDE‐154^a^
Contemporary: 2006–2017	All water types	↓3.8	↓6.5	↓5.6	↓6.4	NS	NS
	Great Lakes	↓7.3	↓9.1	↓6.4	↓8.1	NS	NS
	Rivers	↓3.2	↓6.9	↓6.4	↓8.1	NS	NS
	Inland lakes	↑32.7	↑88	↓6.4	↓8.1	NS	NS
Long term: 1995–2015	Lake Superior	↓3.3	Not assessed				

^a^ NS = no significant trend found for these congeners.

PBDE = polybrominated diphenyl ether.

**Figure 4:**
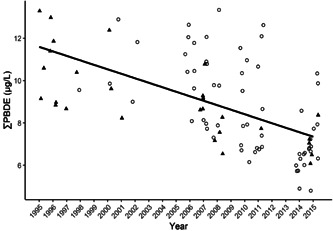
Sum polybrominated diphenyl ethers (∑PBDEs) in bald eagle (*Haliaeetus leucocephalus*) nestling plasma at the Lake Superior study areas showing a 3.3% annual decline. Solid triangles = Lake Superior south shore (LSSS); open circles = Apostle Islands (APIS). We found no difference between study areas or between pre‐ or post‐2004, when penta‐PBDE production was reduced.

We examined long‐term trends (1995–2015) in ∑PBDEs on Lake Superior study areas (Apostle Islands and Lake Superior southern shore) and found no difference in slope between the pre‐ and post‐2004 reduction in penta‐PBDE production (*β* = 0.0211, *t* = –0.35, *p* = 0.73) and no significant difference in trend between the 2 study areas (second‐best model included study area, AIC wt = 0.18, *t* = –0.006, *p* = 0.99). The selected model (wt = 0.57) included year and age as fixed effects and indicated a 3.3% decline in ∑PBDEs across both Lake Superior study areas annually (*β* = –0.0144 on log_10_ scale, *t* = –3.54, *p* = 0.001; Table 2 and Figure 4).

Congener BDE‐47 declined by 6.5% annually in nestling plasma across all study areas (*β* = –0.0636, χ^2^ = 70.08, *p* < 0.001); declined by 9.1% in Great Lakes nestlings (*β* = –0.0867); declined by 6.9% in nestlings on rivers (*β* = –0.0668); and increased by 88% annually in nestlings from the inland lakes study area (*β* = 0.6319), although, as stated earlier, this study area had relatively few samples over only 2 yr (Figure 3 and Table 2).

Congener BDE‐99 also declined: 5.6% annually in nestling plasma across all study areas (*β* = –0.0548, χ^2^ = 41.22, *p* < 0.001) and declined by 6.4% annually for all waterbody types (second‐best model included only year, ΔAIC*_c_* = 23.6, wt = 0, *β* = –0.0622, χ^2^ = 55.1, *p* < 0.001). Similarly, concentrations of BDE‐100 declined across all study areas by 6.4% annually (*β* = −0.0623, χ^2^ = 52.59, *p* < 0.001) and 8.1% annually across all waterbody types (best model included only year; *β* = –0.0775, χ^2^ = 101.9, *p* < 0.001; Figures 2 and 3).

We did not detect statistically significant trends in BDE‐153 or ‐154 (model with study area and year had no support, AIC*_c_* wt = 0).

## DISCUSSION

### Concentrations

We found the highest concentrations of PBDEs in bald eagle nestlings near urban centers (e.g., Minneapolis/St. Paul, MN, USA) and along the shore of the Great Lakes (Figure 1). We found the lowest concentrations in sparsely populated areas (upper St. Croix River, Northern Highlands, and lower Wisconsin River). This agrees with our previous analysis (Route et al. 2014a) and other studies showing higher PBDE concentrations in air, water, and biota near large population centers (Henny et al. 2009; Chen and Hale 2010; Spears and Isanhart 2014; Ruge et al. 2015), and that the Great Lakes receive pollution from urban centers spread across a large region. Urban areas have several sources of PBDEs, including large municipal wastewater treatment plants (WWTPs; Melymuk et al. 2014), landfills (Route et al. 2014a), and industries that manufacture products containing PBDEs (Rahman et al. 2001). Municipal WWTPs in particular have been implicated as major sources of PBDEs that are released into the waste stream from textiles and other products from homes, commercial businesses, and industry (North 2004; Song et al. 2006). Municipal WWTPs are sources of other persistent chemicals in this region as well, including perfluorinated compounds (Route et al. 2014b; Dykstra et al. 2020) and pharmaceuticals and personal care products (Elliott et al. 2018). Another potential source associated with more populated areas is discarded plastics (litter) that end up in the region's rivers and lakes (Baldwin et al. 2016) where they are abraded by wave action and leached of chemical constituents including PBDEs (Rochman et al. 2014). Unlike other chemicals that are bonded to the polymer, PBDEs are blended into plastics as additives and are easily leached out (Costa et al. 2008).

Eagle nestlings in the Great Lakes study areas had significantly higher concentrations of ∑PBDEs than nestlings at inland lakes but not rivers (Figure 1). Our inland lakes study area (Northern Highlands) is in a remote region, so the low concentrations there are likely due to few sources of PBDEs. In contrast, we attribute the high levels of PBDEs on the Great Lakes to: aerial deposition of PBDEs onto their large surface areas from sources inside and outside of the region; lake currents that can carry suspended PBDEs from nearby and distant point sources; water transport of PBDEs from sources along tributaries to the Great Lakes; and the slow flushing and degradation of PBDEs in the slow‐flowing and relatively cool waters (Route et al. 2014a; Dykstra et al. 2020). However, the river study areas spanned from 2 with the highest PBDE concentrations (Mississippi River, Pools3+4) that were influenced by Minneapolis/St. Paul, to 2 with the lowest concentrations (upper St. Croix River and lower Wisconsin River) in sparsely populated rural areas (Figure 1). Although we did not find statistical differences between individual study areas—likely due to small sample size in some areas—our data suggest that nestlings along the Wisconsin River have lower concentrations of PBDEs than those on the lower St. Croix River and the section of the Mississippi River we sampled (Figure 1). Differences in concentrations in nestlings are likely due to their proximity to PBDE sources, but could also be influenced by the type of prey and their trophic position in the food web (see *PBDE trends* section). The rivers, therefore, represent a cross section of PBDE pollution as they meander through urban and rural landscapes.

### Congener profiles

Bald eagle nestlings from all study areas had congener profiles that were typical for top‐level predators of Midwestern large rivers (Blocksom et al. 2010) and the Great Lakes (Gauthier et al. 2008; Pérez‐Fuentetaja et al. 2015; Gandhi et al. 2017; Su et al. 2017; Zhou et al. 2019), although there was substantial variation. We found BDE‐47 to be consistently highest, followed by BDEs‐99 and ‐100, whereas BDE‐153 and ‐154 contributed much less across all 12 study areas (Table 1). In the upper Mississippi River these same 5 congeners were more abundant in large predatory fish than in small prey fish (Blocksom et al. 2010). These 5 congeners were found at higher levels in great blue heron (*A. herodias*) eggs from a heron rookery on the Mississippi River near the municipal WWTP in St. Paul (center of our Mississippi River study area) than at heron rookeries up river or down river, again suggesting proximity to source as a factor (Custer et al. 2010). Along with BDE‐49, these 5 congeners made up 94% of the ∑PBDEs in Caspian tern (*Hydroprogne caspia*) eggs from the Great Lakes (Su et al. 2017). Eggs of herring gulls (*Larus argentatus)* from the Great Lakes also had mainly BDE‐47, ‐99, ‐100, and ‐153 (Su et al. 2015).

For consistency with past studies, we assessed ∑PBDEs of 9 congeners with individual attention to the 5 most prevalent. However, in 2010 we began measuring 8 additional congeners and found 3 of them (BDE‐28, ‐49, and ‐66) to have geometric means as high as 0.75 µg/L (range = LOQ–5.3 µg/L; Supplemental Data, Table S4). These concentrations are low, but in several cases the estimated means exceeded those of the 5 congeners we assess in detail, particularly BDE‐153 and ‐154. We did not examine these minor congeners individually due to the large proportion (>60%) of samples <LOQ (Helsel 2012). Nonetheless, the cumulative concentrations of these infrequently detected congeners may be important in some study areas. For example, lower brominated congeners such as BDE‐28, ‐49, and ‐66 are more persistent and thought to be more toxic than the higher brominated congeners (US Agency for Toxic Substances and Disease Registry 2017). We found little evidence of PBDE congeners BDE‐156 and higher in nestling plasma with the exception of the Green Bay/Lake Michigan study area, where measurable concentrations of these highly brominated congeners were found in 1 to 5 samples (11 total occurrences; Supplemental Data, Table S3). We found only one sample from Green Bay/Lake Michigan with concentrations of BDE‐209 >LOQ, although the laboratory's high LOQ of 10.0 µg/L for BDE‐209 leaves open the possibility that this congener may have been present at low concentrations in more samples.

### PBDE trends

Previously, we reported a 5.5% annual decline in ∑PBDEs from 2006 to 2011 in 6 of these same study areas (Route et al. 2014a). The present study includes 5 more yr and 6 additional study areas showing a sustained, although somewhat slowed, rate of decline (3.8% annually) across the larger region. However, ∑PBDE trends varied and declined more steeply (7.3%) in nestlings from the combined Great Lakes compared with river study areas (3.2%). Moreover, as stated in *Materials and Methods*, Batterman et al. (2007) found loss rates of 1 to 2% annually for PBDEs in frozen archives of fish tissue, suggesting that our frozen plasma samples for Lake Superior nestlings (1995–2001, *n* = 18; Supplemental Data, Table S1) could have experienced some loss during the 10 to 20 yr they remained frozen before laboratory analysis. Hence, the 3.3% annual rate of decline we found in the Lake Superior bald eagle nestlings from 1995 through 2015 should be considered a minimum. As expected, the ∑PBDE declines mirror those of the 3 most abundant congeners (BDE‐47, ‐99, and ‐100) in nestling plasma (6.4–9.1%) across most study areas.

The declines we observed in nestlings from the Great Lakes are likely most indicative of regional PBDE trends, because the Great Lakes, with their large surface areas and drainage basins, integrate air‐borne and land‐based pollutants across a large area (combined drainage area = 246 000 km^2^; Hites 2006). Ruge et al. (2015) found that PBDEs also declined in both air and water around Lake Superior from 2005 to 2011, and that the PBDE congener mix in air and water was dominated by BDE‐47, followed by BDE‐28, ‐49, ‐99, and ‐100, whereas BDE‐153 was low and BDE‐154 was rarely detected. Similar to our findings, Ruge et al. (2015) concluded that PBDE concentrations in air and water were highest near urban and industrial centers and that the most common congener, BDE‐47, underwent net deposition (movement from air to water) at sample sites near urban centers and net volatilization (movement from water to air) in remote open water sites. Declines in PBDEs have also been found in lake trout (*Salvelinus namaycush*) and walleye (*Sander vitreus*) beginning in approximately the year 2000 (Crimmins et al. 2012; Gandhi et al. 2017). Concentrations in fillets of 18 fish species decreased or plateaued between 2006 and 2012 (Crimmins et al. 2012). The PBDEs in eggs of Great Lakes herring gulls reached a plateau by approximately 2000 (Gauthier et al. 2008) and then declined between 2006 and 2012 (Su et al. 2015). In contrast with these declines, we found no statistically significant trend in BDE‐153 and ‐154 in nestling plasma among study areas or water types, and we found increases in both ∑PBDE and BDE‐47 in plasma from nestlings at inland lakes, although few samples (*n* = 25) and short time‐span for this result suggests additional study is needed at inland lakes. Visual inspection of our data in Figure 2 suggests that, although we did not find statistically significant trends in BDE‐153 and ‐154, these 2 congeners likely also declined. The lack of a statistically significant trend could be due to lower sample size for BDE‐153 (all 5 Northern Highlands samples were <LOQ and were dropped from trend analyses) and/or high variability (e.g., concentrations of both BDE‐153 and ‐154 were highly variable in study area Fox River/Green Bay; Figure 2).

These declines in air, water, and biota are consistent with global reduction in the penta‐ and octa‐PBDE production in the early 2000s. Sediments, however, may hold deposits of PBDEs for many years. For example, Li et al. (2006) found that PBDEs in sediments increased in all 5 Great Lakes from the 1970s to 2002, with longer doubling times than for fish and humans, suggesting a lag in the movement of PBDEs to sediments in the region. Moreover, lake and stream bottom‐sediments can serve as a reservoir of higher brominated PBDE congeners that can be biotransformed to lower brominated congeners. For example, there is evidence that deca‐PBDEs, largely consisting of BDE‐209, can slowly debrominate in sediments of boreal lakes (Orihel et al. 2016) and that microbes in sediments may be involved in debrominating deca‐BDEs to octa‐ (including BDE‐153), and even to penta‐BDEs (such as BDE‐99; He et al. 2006). Viganò et al. (2012) also concluded that debromination of BDE‐209, which they found at high levels in river sediments, resulted in novel congeners BDE‐179, ‐188, and ‐202 in cyprinid fishes (mainly common carp [*Cyprinus carpio*]). These congeners are not found in technical PBDE formulations, but are known byproducts of BDE‐209 debromination (Viganò et al. 2012). This biotransformation of BDE‐209 to lower brominated congeners has also been documented in lake trout from the Great Lakes, although models suggest that this process is slow, with low to moderate transformations to BDE‐47 and ‐99 over 15 yr (Gandhi et al. 2011). As an aside to this region‐wide analysis, we found ∑PBDEs increased at the upper St. Croix River study area. In Figure 3 we distinguish upper St. Croix River (open blue squares) from other river study areas (filled blue squares) to show the consistently low levels at upper St. Croix River, with a notable increase over time (see also Figure 2A). In a test of marginal means between 3 2‐yr sampling periods, we found that ∑PBDEs increased significantly (*p* < 0.001; Supplemental Data, Figure S1), and amounted to a nearly 2‐fold increase from 2006 to 2015. Both upper St. Croix River and the inland lakes study area (Northern Highlands) where we found increases are located in the remote, northern forested zone of Wisconsin, and we know of no major point sources for PBDEs in this area. Further research is needed to understand whether debromination of higher brominated congeners (e.g., BDE‐209) to lower brominated congeners might contribute to the lack of significant declines in BDE‐153 and ‐154 and/or the significant increases in ∑PBDEs we found at upper St. Croix River and the inland lakes study area.

Others have noted evidence that PBDE profiles have transitioned toward more highly brominated congeners in some prey fish (Crimmins et al. 2012), possibly because of industry switching to the higher brominated congeners after penta‐ and octa‐PBDE production was reduced (Ross et al. 2009). This is consistent with our findings of decreases in lower brominated congeners (BDE‐47, ‐99, and ‐100) in nestlings and a lack of significant trends in the higher brominated congeners (BDE‐153 and ‐154). The PBDEs in lake and stream sediments, including the Great Lakes, are dominated by the highly brominated congener BDE‐209 (Li et al. 2006; Akortia et al. 2016) found in deca‐BDE products (de Wit 2002). Some fish in lower trophic levels in the Great Lakes are also dominated by BDE‐209 (Gandhi et al. 2017). We speculate that a reservoir of higher brominated congeners in the lower food web and bottom‐sediments, which are rarely found in bald eagle plasma because they do not readily bioaccumulate, could be a source of lower brominated congeners to eagles through biotransformation. Furthermore, this transformation could have a greater effect in systems that are less contaminated, such as upper St. Croix River. That is, in areas of high contamination, such as Mississippi River, the industry‐wide reduction in production of PBDEs would cause a relatively immediate decrease in bioavailability of all PBDE congeners. However, in areas of low contamination, complex metabolic processes that transform higher to lower brominated congeners would be of proportionally greater importance. For example, carp (*Cyprinus* spp.) metabolize higher brominated BDE‐183 and ‐203 to form BDE‐153, which in turn is metabolized by carp to BDE‐47; however, carp do not readily metabolize BDE‐47, thus causing a net increase in this lower brominated congener (Roberts et al. 2011). Rainbow trout (*Oncorhynchus mykiss*), on the other hand, slowly transform BDE‐99 and ‐153, and minimally metabolize BDE‐209 (Kierkegaard et al. 1999; Roberts et al. 2011). Other research has also found that BDE‐209 debrominates to lower brominated congeners in lake trout (Gandhi et al. 2011).

Bald eagles are well known for preying and scavenging on fish and other aquatic animals (Kozie and Anderson 1991; Watson 2002). In a subsample of 317 nests in 6 of our study areas (Apostle Islands, lower St. Croix River, Lake Superior southern shore, Mississippi River, Pools3+4, and upper St. Croix River) we found 80.4% of nests contained remains of fish, 29.3% remains of birds, 25.6% remains of mammals, and 19.9% remains of reptiles (Supplemental Data, Table S3). These frequencies do not necessarily represent the importance of each prey type because we could not determine the quantity of flesh consumed, and prey remains generally over‐represent taxa such as birds (Kozie and Anderson 1991). Nonetheless, the prey remains do reflect the eagle's dependence on the aquatic food web. Moreover, we found the frequency of each prey type differed between waterbody type. Nests on the Great Lakes (Apostle Islands and Lake Superior southern shore) had nearly equal frequencies of fish and bird remains (64.3 and 63.4%, respectively), whereas nests on rivers (lower St. Croix River, Mississippi River, Pools3+4, and upper St. Croix River) more frequently contained fish and had a greater variety of other taxon (83.9% fish, 51.1% birds, 58.8% mammal, and 46.6% reptile; Supplemental Data, Table S3). The relatively high frequency of birds in the diet of bald eagles on the Great Lakes has also been documented by Kozie and Anderson (1991) and Dykstra et al. (2001, 2005a, 2005b), who argued that most birds fed on by eagles are themselves high on the aquatic food chain, thereby increasing the effect of biomagnification. Thus, the assemblage of aquatic prey, their relative trophic level, and their metabolic activities likely influence the PBDE congener profiles and trends that are subsequently measured in bald eagle nestling plasma.

## CONCLUSIONS

We conclude that most PBDE congeners that bioaccumulate in bald eagles have declined, and that the decline is consistent with the reduction in penta‐ and oct‐PBDE use that began in the mid‐2000s. We found that nestlings in rural areas had the lowest concentrations of PBDEs and that nestlings near urban centers had the highest concentrations, likely due to the greater abundance of PBDE sources in more human‐dominated landscapes. The declines we observed were not universal, however. Some of the more remote study areas, with comparatively low concentrations, exhibited increases in some congeners. The overall concentrations and patterns in congener profiles we found in bald eagle nestling plasma reflect a complex mix of factors, including: their availability in the environment through source pollution; the chemical properties of each congener that make them more or less likely to persist, be absorbed in tissues, and bioaccumulate up the food chain; the species and trophic position of the prey available to eagles; and the physical and biological characteristics of waterbodies, which can modulate PBDE transport, deposition, and degradation.

We have no reason to suspect that the PBDE concentrations we found negatively affected bald eagle productivity in this region because study areas with the highest eagle productivity (e.g., Mississippi River and lower St. Croix River) also have high PBDE concentrations whereas upper St. Croix River, with low bald eagle productivity, has low PBDE concentrations (Route et al. 2011). Similar conclusions have been reached for PCBs in some of these same eagle populations (Dykstra et al. 1998, 2019). Nonetheless, the patterns of contamination we found are instructive for the public and regulators concerned with human health considerations such as fish consumption advisories. Bald eagles are similar to humans in that both are tertiary predators in aquatic systems, and hence the patterns we observed in nestlings may be indicative of those in humans who consume fish from the same waterbodies. Also, although we did not detect adverse effects on eagle productivity, the nervous system and endocrine system are particularly sensitive targets of the lower brominated congeners (US Agency for Toxic Substances and Disease Registry 2017), and the long‐term effects of chronic exposure to these and other physiological and behavioral systems were beyond the scope of the present study.

The variation in PBDE contamination among study areas and waterbody type, and the increasing trends in some areas, suggest that continued monitoring is warranted. More in‐depth research into the fate of these compounds, especially how congeners are metabolized and biotransformed, could help clarify the differences in concentrations and trends we found. Finally, our broad, landscape‐scale assessment highlights the importance of conducting multisite, long‐term monitoring when assessing trends. Such monitoring should be designed to allow fine‐scale study area–specific analyses to reveal local trends as well.

## Supplemental Data

The Supplemental Data are available on the Wiley Online Library at https://doi.org/10.1002/etc.5006.

## Disclaimer

The authors declare no competing financial interests.

## Author Contributions Statement

W. Route, S. Strom, and M. Meyer conceived the work, developed sampling protocols, and collected the data; K. Williams and C. Dykstra conducted summary and statistical analyses; all authors contributed to writing the manuscript and have approved the final version.

## Supporting information

This article includes online‐only Supplemental Data.

Supporting information.

## Data Availability

Data for this manuscript that were collected within the boundaries of federally managed areas are publicly available free of charge from the National Park Service Data Store at: https://irma.nps.gov/DataStore/PBDEs. Data, associated metadata, and calculation tools are also available from the corresponding author (billroute2@gmail.com).
