# Supplementary material for: Patterns and Trends of Polybrominated Diphenyl Ethers in Bald Eagle Nestlings in Minnesota and Wisconsin, USA
Source: Environ Toxicol Chem. 2021 Mar 10;40(6):1606–18. doi: 10.1002/etc.5006 (PMC8252721; doi:10.1002/etc.5006)
Supplement: Supplementary file 1 — Supporting information. [file ETC-40-1606-s001.docx]

**Supporting Information.** The following material is in support of: *Patterns and Trends of Polybrominated Diphenyl Ethers in Bald Eagle Nestlings in Minnesota and Wisconsin, USA.*

William T. Route, Cheryl R. Dykstra, Sean M. Strom, Michael W. Meyer, and Kelly A. Williams. ____________________________________________________________________________

**List of Tables and Figures:**

**Figure SI1.** Map showing location of study areas where bald eagle (*Haliaeetus leucocephalus*) nestlings were sampled to measure concentrations of PBDEs.

**Table SI1**. Sampling effort and percent of samples below the laboratory’s limits of quantification (LOQ) for each study area.

**Table SI2**. Laboratory quality assurance data, limits of quantification (LOQ), number of sample, and percent samples above LOQ for 17 PBDE congeners measured in this study.

**Table SI3**. Percent censored, range, and study area of detection for 12 PBDE congeners not reported in detail in the manuscript.

**Table SI4**. Geometric means and range of three PBDE congeners with >10% of samples above LOQ and not reported in detail in the manuscript.

**Table SI5**. Frequency of occurrence for prey items found in bald eagle nests.

**Figure SI2.** Changing levels of ∑PBDEs for study area USACN across three time periods.

**8 pages total**


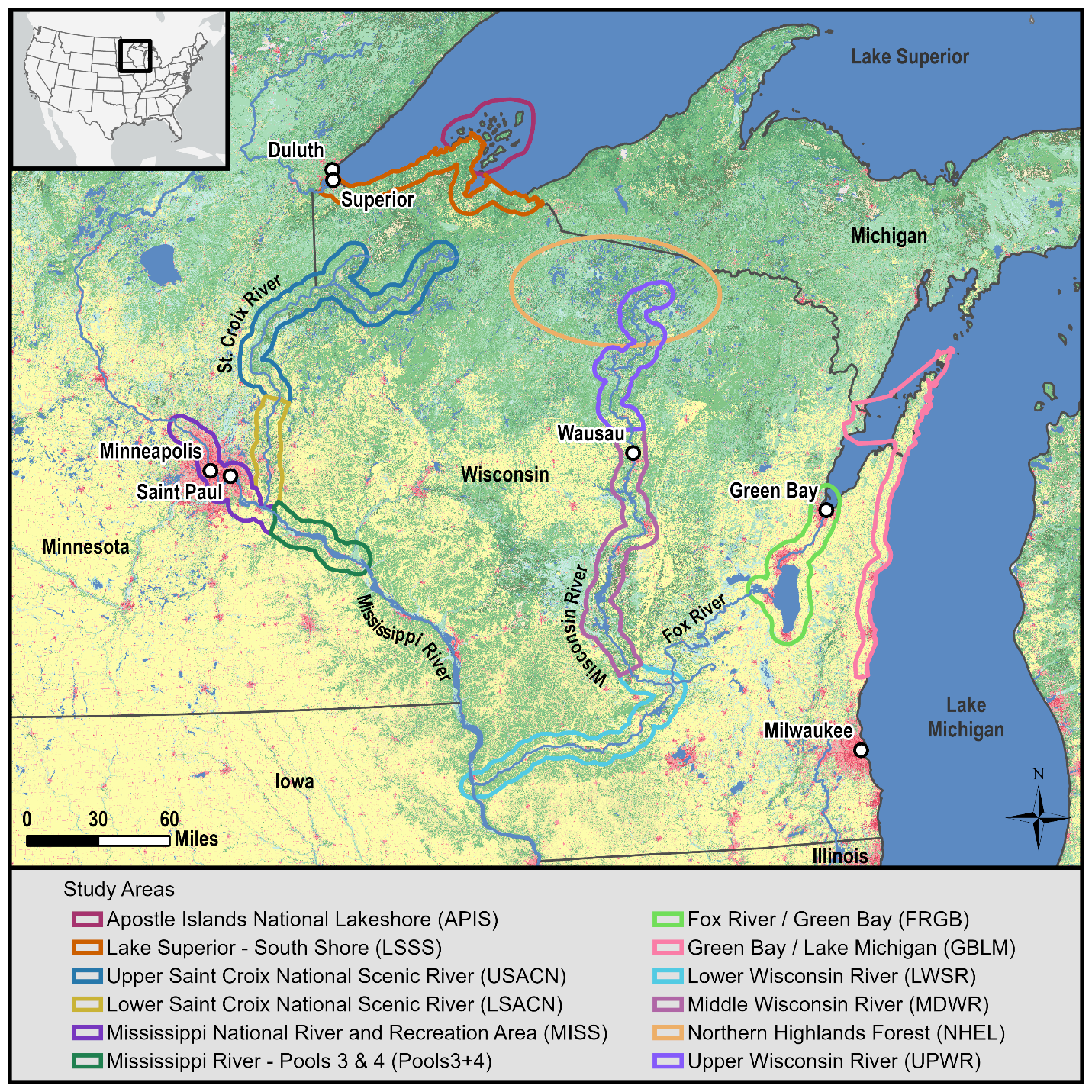


**Figure SI1.** Names, acronyms, and locations of 12 study areas where bald eagle nestlings were sampled for PBDEs in Minnesota and Wisconsin, USA. Background layer is from the USGS National Land Cover Database; dark green = conifer, light green = mixed hardwood, light yellow = mixed crop lands and shrub, light pink to red = increasing intensity of human development (more information available at <https://www.usgs.gov/centers/eros/science/national-land-cover-database>).

**Table SI1.** Years sampled, number of bald eagle territories sampled, number of nestling plasma samples measured for PBDEs, and the mean, minimum, and maximum percent of 17 PBDE congeners that were below the laboratory’s limits of quantification (LOQ) per study area.

|  |  |  |  | **% congeners <LOQ/sample** | | |
| --- | --- | --- | --- | --- | --- | --- |
| **study area^a^** | **years sampled** | **territories^b^** | **samples^c^** | **mean** | **minimum** | **maximum** |
| APIS | 1998-2008; 2010, 2011, 2014, 2015 | 27 | 63 | 58.3% | 22.2% | 82.4% |
| FRGB | 2011-2013; 2016 | 12 | 23 | 80.6% | 70.6% | 94.1% |
| GBLM | 2011-2013; 2016 | 19 | 30 | 73.1% | 52.9% | 88.2% |
| LSACN | 2006-2011; 2014, 2015 | 22 | 65 | 59.0% | 22.2% | 88.2% |
| LSSS | 1995-2001; 2007, 2008, 2011, 2015 | 20 | 29 | 55.7% | 11.1% | 82.4% |
| LWSR | 2015 | 5 | 5 | 82.4% | 76.5% | 88.2% |
| MDWR | 2015 | 7 | 7 | 81.5% | 64.7% | 94.1% |
| MISS | 2006-2011; 2014, 2015 | 46 | 139 | 52.8% | 22.2% | 76.5% |
| NHEL | 2014, 2017 | 24 | 25 | 86.8% | 70.6% | 100.0% |
| Pools3+4 | 2008-2011 | 21 | 33 | 41.7% | 22.2% | 64.7% |
| UPWR | 2014, 2015, 2017 | 7 | 8 | 80.2% | 70.6% | 94.1% |
| USACN | 2006, 2007, 2010, 2011, 2014, 2015 | 31 | 65 | 78.4% | 33.3% | 94.1% |
| **totals** |  | **241** | **492** | **69.2%** | **11.1%** | **100.0%** |

^a^ = Study areas included Apostle Islands National Lakeshore (APIS), Lake Superior’s southern shore in Wisconsin (LSSS), upper St. Croix National Scenic Riverway (USACN), lower St. Croix National Scenic Riverway (LSACN), Mississippi National River and Recreation Area (MISS), Pools 3 and 4 of the Mississippi River (Pools3+4), Fox River and lower Green Bay (FRGB), upper Green Bay and Lake Michigan (GBLM), upper Wisconsin River (UPWR), middle Wisconsin River (MDWR), lower Wisconsin River (LWSR), and Northern Highlands American Legion State Forest (NHEL).

^b^ = Known, named territories; excludes 1 unidentified territory at MISS for which 1 sample was collected.

^c^ = Includes 18 archived samples: 5 at APIS and 13 at LSSS.

**Table SI2.** Number of quality assurance (QA) samples, average percent recovery of QA samples, the laboratory’s limits of quantification (LOQ), number of samples measured, and the number and percent of those samples that were ≥LOQ for 17 PBDE congeners. Table is sorted by % ≥ LOQ to highlight the relative frequency of detection.

| **PBDE congener^a^** | **lab QA samples^b^** | **lab % recovery^b^** | **lab LOQ^b^** | **samples measured^c^** | **no. ≥ LOQ^d^** | **% ≥ LOQ^d^** |
| --- | --- | --- | --- | --- | --- | --- |
| BDE-47* | 32 | 90.1 | 0.2 | 474 | 472 | 99.6 |
| BDE-99* | 32 | 94.4 | 0.2 | 474 | 439 | 92.6 |
| BDE-100* | 32 | 100.7 | 0.2 | 474 | 427 | 90.1 |
| BDE-153* | 32 | 100.9 | 0.4 | 474 | 297 | 62.7 |
| BDE-154* | 32 | 100.4 | 0.4 | 474 | 292 | 61.6 |
| BDE-28* | 32 | 97.7 | 0.2 | 474 | 283 | 40.3 |
| BDE-49 | 14 | 88.5 | 0.2 | 321 | 86 | 26.8 |
| BDE-66* | 32 | 95.3 | 0.2 | 474 | 56 | 11.8 |
| BDE-85* | 32 | 97.3 | 0.2 | 474 | 11 | 2.3 |
| BDE-197 | 14 | 89.6 | 0.4 | 321 | 5 | 1.6 |
| BDE-138* | 32 | 103.2 | 0.4 | 474 | 4 | 0.8 |
| BDE-196 | 14 | 86.9 | 0.4 | 321 | 2 | 0.6 |
| BDE-207 | 14 | 80.2 | 0.4 | 321 | 2 | 0.6 |
| BDE-183 | 14 | 84.9 | 0.4 | 321 | 1 | 0.3 |
| BDE-209 | 14 | 98.0 | 10.0 | 321 | 1 | 0.3 |
| BDE-156 | 14 | 88.8 | 0.4 | 321 | 0 | 0.0 |
| BDE-206 | 14 | 98.0 | 0.4 | 321 | 0 | 0.0 |

^a^ = Congeners with * were included in ∑PBDE calculations for consistency with past studies.

^b^ = From quality assurance report available from the US National Park Service on samples collected 2006-2011: number of quality assurance samples tested, average percent recovery of known quantities of each congener, and the laboratory’s limits of quantification.

^c^ = Cumulative number of samples measured for this congener in this study across all 12 study areas, 1995-2017.

^d^ = Number of samples that were at or above the limits of quantification and the average percent for each congener.

**Table SI3.** Percent of total samples < LOQ, range (not Kaplan-Meier adjusted), and study areas where they were detected for 12 PBDE congeners not reported in detail in the manuscript. Table is sorted by % < LOQ.

| **congener^a^** | **% < LOQ** | **concentration range** | **samples above LOQ for each study area^b^** |
| --- | --- | --- | --- |
| BDE-28 | 59.7 | LOQ – 2.60 | 15 APIS, 6 LSSS, 6 USACN, 38 LSACN, 103 MISS, 18 Pools3+4, 1 FRGB, 3 GBLM, 1 MDWR |
| BDE-49 | 73.2 | LOQ – 1.50 | 21 APIS, 6 USACN, 16 LSACN, 41 MISS |
| BDE-66* | 88.2 | LOQ – 0.480 | 13 APIS, 4 LSSS, 4LSACN, 24 MISS, 11 Pools3+4 |
| BDE-85* | 97.7 | LOQ – 0.610 | 1 APIS, 7MISS, 3 MDWR |
| BDE-197 | 98.4 | LOQ – 0.800 | 5 GBLM |
| BDE-138* | 99.2 | LOQ – 3.30 | 1 APIS & LSSS, 2 GBLM |
| BDE-196 | 99.4 | LOQ – 0.650 | 2 GBLM |
| BDE-207 | 99.4 | LOQ – 4.40 | 2 GBLM |
| BDE-183 | 99.7 | LOQ – 0.400 | 1 GBLM |
| BDE-209 | 99.7 | LOQ – 18.0 | 1 GBLM |
| BDE-156 | 100 | < LOQ |  |
| BDE-206 | 100 | < LOQ |  |

^a^ = Congeners with * were included in ∑PBDE calculations in the manuscript for consistency with past studies. ^b^ = Study areas: Apostle Islands National Lakeshore (APIS), Lake Superior’s southern shore in Wisconsin (LSSS), upper St. Croix National Scenic Riverway (USACN), lower St. Croix National Scenic Riverway (LSACN), Mississippi National River and Recreation Area (MISS), Pools 3 and 4 of the Mississippi River (Pools3+4), Fox River and lower Green Bay (FRGB), upper Green Bay and Lake Michigan (GBLM), upper Wisconsin River (UPWR), middle Wisconsin River (MDWR), lower Wisconsin River (LWSR), and Northern Highlands American Legion State Forest (NHEL).

**Table SI4.** Geometric means and range of three PBDE congeners (ug/L) measured 2010 – 2017 at 12 study areas in Wisconsin and Minnesota. These three congeners are novel to our long-term monitoring (1995-2017) and are not reported in detail in the manuscript. Means are estimated from frailty models using the Kaplan-Meier (KM) procedure to replace results below LOQ (see methods in manuscript), however, we did not calculate means when a congener at a study area had <10% of samples above LOQ. Range is the high and low measurements of raw data.

|  |  | **mean and (range) in µg/L** | | |
| --- | --- | --- | --- | --- |
| **study area** | **total n** | **BDE-28** | **BDE-49** | **BDE-66** |
| APIS | 58 | 0.150  (LOQ-1.60) | 0.270  (LOQ-1.40) | 0.160  (<LOQ-0.430) |
| LSSS | 16 | 0.280  (LOQ-2.00) | (LOQ-0.420) | 0.150  (<LOQ-0.330) |
| USACN | 65 | 0.080  (<LOQ-0.260) | 0.150  (LOQ-0.440) | (<LOQ-0.200) |
| LSACN | 65 | 0.330  (LOQ-4.10) | 0.590  (0.280-1.98) | 0.120  (<LOQ-0.400) |
| MISS | 139 | 0.430  (LOQ-5.30) | 0.500  (LOQ-1.97) | 0.140  (<LOQ-0.480) |
| Pools3+4 | 33 | 0.750  (0.340-1.90) | (1.01-1.56)^a^ | 0.140  (<LOQ-0.400) |
| FRGB | 23 | 0.080  (<LOQ-1.30) | (<LOQ-2.35) | (<LOQ-0.300) |
| GBLM | 30 | 0.070  (<LOQ-0.400) | (<LOQ-1.04) | (<LOQ-0.200) |
| UPWR | 8 | (LOQ-0.200) | (<LOQ-0.150) | (<LOQ-0.180) |
| MDWR | 7 | 0.080  (<LOQ-0.250) | (<LOQ-0.200) | (<LOQ-0.200) |
| LWSR | 5 | (<LOQ-0.180) | (<LOQ-0.180) | (<LOQ-0.150) |
| NHEL | 25 | (<LOQ-0.200) | 0.100  (<LOQ-0.240) | (<LOQ-0.200) |

a = Possible interference with another chemical noted for this congener.

**Table SI5.** Frequency of occurrence (*f*) and percent (%) of bald eagle nests that contained evidence of four major taxon of prey at six study areas in Minnesota and Wisconsin, USA. Nests were checked during routine sampling of nestlings from 2006-2015 for monitoring the concentrations of environmental contaminants.

|  | **study area^a^** | | | | | | | | | | | |
| --- | --- | --- | --- | --- | --- | --- | --- | --- | --- | --- | --- | --- |
|  | **APIS** | | **LSSS** | | **MISS** | | **Pools3+4** | | **LSACN** | | **USACN** | |
| **prey item** | ***f*** | **%** | ***f*** | **%** | ***f*** | **%** | ***f*** | **%** | ***f*** | **%** | ***f*** | **%** |
| fish | 27 | 65.9 | 9 | 60.0 | 107 | 81.7 | 17 | 85.0 | 44 | 88.0 | 51 | 85.0 |
| bird | 20 | 48.8 | 6 | 40.0 | 37 | 28.2 | 1 | 5.0 | 16 | 32.0 | 13 | 21.7 |
| mammal | 1 | 2.4 | 3 | 20.0 | 33 | 25.2 | 5 | 25.0 | 13 | 26.0 | 26 | 43.3 |
| reptile | 1 | 2.4 | 1 | 6.7 | 19 | 14.5 | 13 | 65.0 | 12 | 24.0 | 17 | 28.3 |
|  |  |  |  |  |  |  |  |  |  |  |  |  |
| total nests = | 41 |  | 15 |  | 131 |  | 20 |  | 50 |  | 60 |  |

^a^ = Study areas: Apostle Islands National Lakeshore (APIS), Lake Superior’s southern shore in Wisconsin (LSSS), upper St. Croix National Scenic Riverway (USACN), lower St. Croix National Scenic Riverway (LSACN), Mississippi National River and Recreation Area (MISS), Pools 3 and 4 of the Mississippi River (Pools3+4).


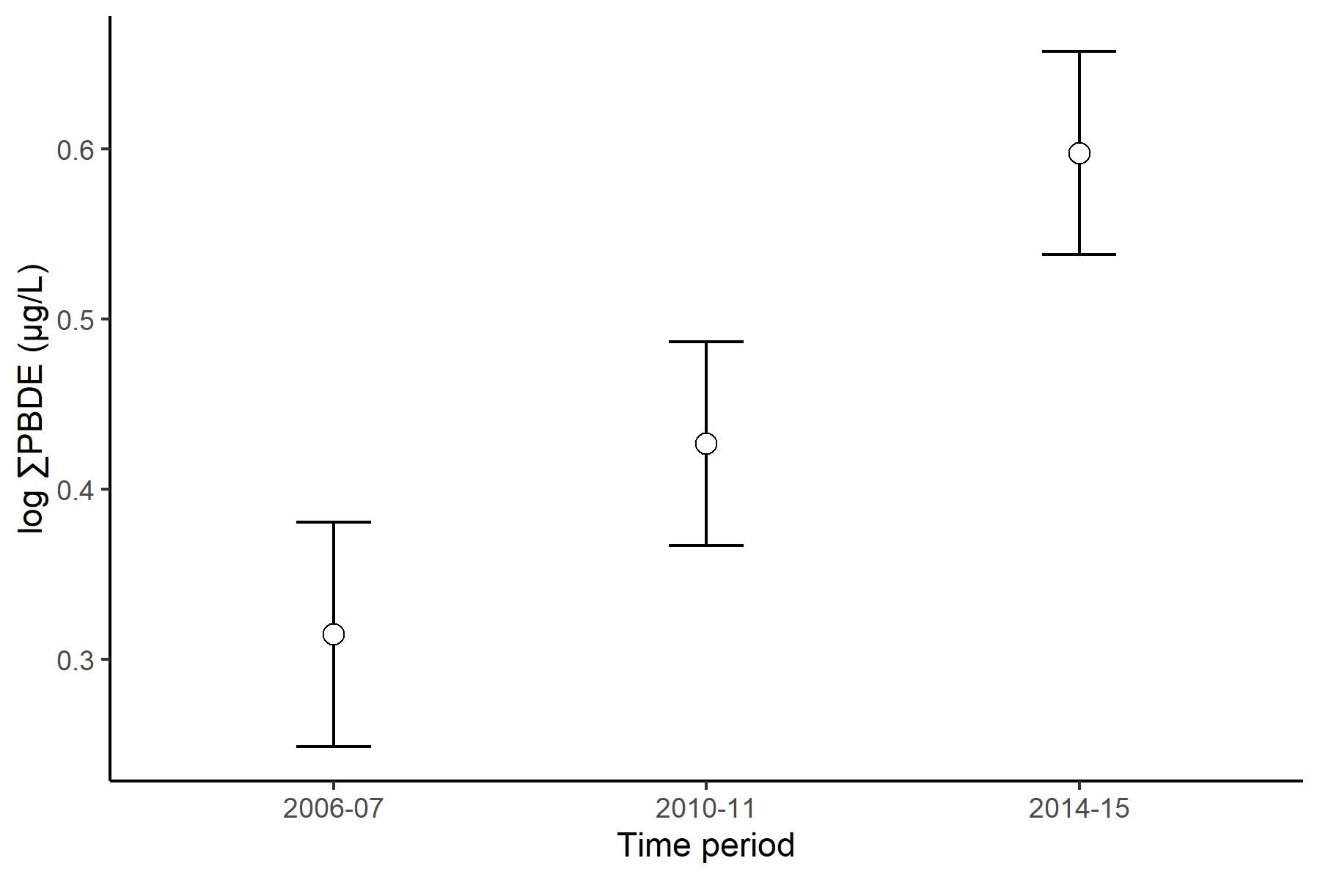


**Figure SI2.**  Changing levels of ∑PBDEs for the upper portion of the St Croix National Scenic Riverway study area (USACN) across three time periods. The charted values are marginal means with SE as 95% confidence intervals; 2006-07, n = 19; 2010-11, n = 23; 2014-15, n = 23. We used a mixed effects model with territory as random effect and time period as a factor to test the null hypothesis that concentrations did not differ among time periods. All time periods differed from each other *p* < 0.001. The lowest concentration of 0.315 ± 0.03 ug/L (log10 scale) was during 2006-2007, increasing to 0.427 ± 0.03 ug/L during 2010-11, and the highest levels were 0.597 ± 0.03 ug/L during 2014-15. The difference constitutes a nearly 2-fold increase from the 2006-07 time period to the 2014-15 time period.
